# Functional analysis of feedback inhibition-insensitive aspartate kinase identified in a threonine-accumulating mutant of *Saccharomyces cerevisiae*

**DOI:** 10.1128/aem.00155-24

**Published:** 2024-03-08

**Authors:** Shota Isogai, Akira Nishimura, Akiko Inoue, Shino Sonohara, Takashi Tsugukuni, Tomoyuki Okada, Hiroshi Takagi

**Affiliations:** 1 Institute for Research Initiative, Nara Institute of Science and Technology, Takayama, Nara, Japan; 2 Plant Bio Business Unit, Musashi Seimitsu Industry Co., Ltd., Toyohashi, Aichi, Japan; Kyoto University, Kyoto, Japan

**Keywords:** yeast, *Saccharomyces cerevisiae*, threonine, aspartate kinase Hom3, allosteric regulation

## Abstract

Humans and mammals need to ingest essential amino acids (EAAs) for protein synthesis. In addition to their importance as nutrients, EAAs are involved in brain homeostasis. However, elderly people are unable to efficiently consume EAAs from their daily diet due to reduced appetite and variations in the contents of EAAs in foods. On the other hand, strains of the yeast *Saccharomyces cerevisiae* that accumulate EAAs would enable elderly people to intakegest adequate amounts of EAAs and thus might slow down the neurodegenerative process, contributing to the extension of their healthy lifespan. In this study, we isolated a mutant (strain HNV-5) that accumulates threonine, an EAA, derived from a diploid laboratory yeast by conventional mutagenesis. Strain HNV-5 carries a novel mutation in the *HOM3* gene encoding the Ala462Thr variant of aspartate kinase (AK). Enzymatic analysis revealed that the Ala462Thr substitution significantly decreased the sensitivity of AK activity to threonine feedback inhibition even in the presence of 50 mM threonine. Interestingly, Ala462Thr substitution did not affect the catalytic ability of Hom3, in contrast to previously reported amino acid substitutions that resulted in reduced sensitivity to threonine feedback inhibition. Furthermore, yeast cells expressing the Ala462Thr variant showed an approximately threefold increase in intracellular threonine content compared to that of the wild-type Hom3. These findings will be useful for the development of threonine-accumulating yeast strains that may improve the quality of life in elderly people.

**IMPORTANCE:**

For humans and mammals, essential amino acids (EAAs) play an important role in maintaining brain function. Therefore, increasing the intake of EAAs by using strains of the yeast *Saccharomyces cerevisiae* that accumulate EAAs may inhibit neurodegeneration in elderly people and thus contribute to extending healthy lifespan and improving their quality of life. Threonine, an EAA, is synthesized from aspartate. Aspartate kinase (AK) catalyzes the first step in threonine biosynthesis and is subject to allosteric regulation by threonine. Here, we isolated a threonine-accumulating mutant of *S. cerevisiae* by conventional mutagenesis and identified a mutant gene encoding a novel variant of AK. In contrast to previously isolated variants, the Hom3 variant exhibited AK activity that was insensitive to feedback inhibition by threonine but retained its catalytic ability. This resulted in increased production of threonine in yeast. These findings open up the possibility for the rational design of AK to increase threonine productivity in yeast.

## INTRODUCTION

As building blocks for protein synthesis, amino acids are fundamental molecules in living organisms. Among the 20 proteinogenic amino acids, nine essential amino acids (EAAs) are nutritionally important for mammals. This is because mammals cannot biosynthesize these amino acids through the endogenous pathway (1). In addition to the roles of EAAs as protein components, recent studies have reported that reduced EAA levels in blood are associated with the development of Alzheimer’s disease in participants with mild cognitive impairment (2, 3) and that EAAs play an important role in maintaining brain homeostasis (4–6). However, it is difficult for elderly people to efficiently consume EAAs from their daily diet due to decreased appetite with aging and variations in EAA contents in foods. The yeast *Saccharomyces cerevisiae* is a generally recognized-as-safe (GRAS) organism and is widely used in the production of nutritional supplements. Therefore, it is expected that *S. cerevisiae* strains that accumulate EAAs would help elderly people ingest an appropriate amount of EAAs, slowing down neurodegeneration and thus contributing to extending their healthy lifespan and improving their quality of life.

**Fig 1:**
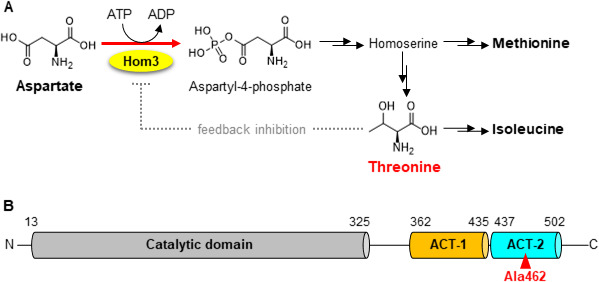
Metabolic pathways of Asp-derived amino acids in the yeast *S. cerevisiae* and domain organization of Hom3. (**A**) Biosynthetic pathways for Asp-derived amino acids. Thr, Met, and Ile are derived from a common precursor Asp. AK catalyzes the first and the rate-limiting step in the biosynthesis of these amino acids and allosterically regulated by Thr. Met biosynthesis is branched from homoserine, and Ile is synthesized from Thr. (**B**) Domain organization of Hom3. Catalytic, ACT domain-1 (ACT-1), and ACT domain-2 (ACT-2) are represented as gray, orange, and light blue bars, respectively. Ala462 is indicated as a red triangle.

Among the nine EAAs, threonine (Thr) is industrially produced using microorganisms and is widely used in foods, feeds, cosmetics, and pharmaceuticals. In *S. cerevisiae*, Thr is biosynthesized from aspartate (Asp) via homoserine in five enzymatic steps. The Thr biosynthetic pathway branches from homoserine to methionine (Met) biosynthesis, where Thr is further converted to isoleucine (Ile) (Fig. 1A) (7). Aspartate kinase (AK), encoded by the *HOM3* gene (8), catalyzes the first reaction of Thr biosynthesis, producing aspartate-4-phosphate by phosphorylation of Asp using ATP. The Hom3-catalyzing reaction is the rate-limiting step in Thr biosynthesis in *S. cerevisiae* because the enzymatic activity of Hom3 is subject to feedback inhibition by the end product Thr (9–14). Based on the amino acid sequence, Hom3 is composed of an N-terminal catalytic domain (gray bar) and two C-terminal regulatory domains (orange and light blue bars) (Fig. 1B). The C-terminal regulatory domains exhibit the characteristic secondary structures of aspartate kinase, chorismate mutase, and the TyrA (ACT) domain, which is widely involved in the allosteric regulation of enzymes responsible for amino acid metabolism (15–17). Previous studies on AKs from various organisms have revealed their structures as bound to substrates and/or inhibitors, including those from *Escherichia coli* (18), *Thermus thermophilus* (19), *Corynebacterium glutamicum* (20), *Clostridium acetobutylicum* (21), *Pseudomonas aeruginosa* (22), *Methanocaldococcus jannaschii* (23), *Arabidopsis thaliana* (24), and *Synechocystis* (Cyanobacteria) (25). Crystal structures of these AKs have shown that the interaction between two ACT domains from different monomers forms the binding sites for the regulatory ligand(s) (26). Several studies have reported the isolation of Thr-accumulating yeast strains by conventional mutagenesis (9, 10, 27) and have identified mutations with amino acid substitutions in the *HOM3* gene of Thr overproducers, such as Glu279Ala (12) and Glu282Asp (14) in the catalytic domain, Ser399Phe (14) in the ACT-1 domain, and Gly452Asp (11) in the ACT-2 domain. Enzymatic analyses revealed that these amino acid substitutions contribute to decreased sensitivity to feedback inhibition by Thr, leading to increased production of Thr. However, the steady-state kinetics of the Glu279Ala (12) and Gly452Asp (13) variants demonstrated that these replacements also affected the kinetic constants, resulting in decreased catalytic ability of Hom3 (Table S1). Hence, amino acid substitutions that are resistant to feedback inhibition while preserving the catalytic ability are sought after to further enhance Thr productivity in yeast.

In this study, we isolated a Thr-accumulating mutant from a diploid laboratory strain of *S. cerevisiae* by conventional mutagenesis and identified a novel amino acid substitution, Ala462Thr, in Hom3. Enzymatic analysis of the Ala462Thr variant Hom3 revealed that the alanine (Ala)-to-Thr replacement at position 462 is desensitized to Thr feedback inhibition even in the presence of 50 mM Thr. Additionally, the Ala462Thr substitution showed no effect on catalytic ability. Furthermore, expression of the Ala462Thr variant Hom3 in *S. cerevisiae* cells increased intracellular Thr content compared to those expressing the wild-type (WT) Hom3.

## RESULTS AND DISCUSSION

### Isolation of yeast mutants with intracellular Thr accumulation

**Fig 2:**
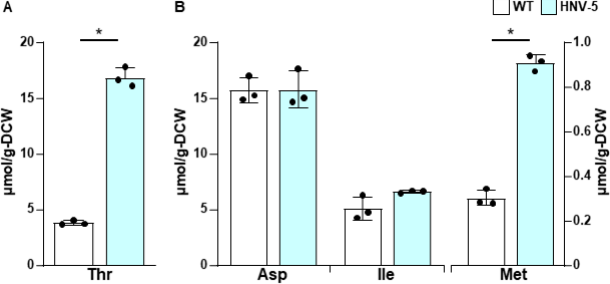
Intracellular amino acid content of a yeast mutant with intracellular Thr accumulation. Strains WT and HNV-5 were cultured in the SD + Am medium for 48 h, and intracellular amino acid contents were measured. Data are presented as means ± standard deviation from three independent experiments. Asterisks indicate statistically significant differences between two strains (Student’s *t* test, *P < 0.05).

We first isolated mutants resistant to hydroxynorvaline (HNV), a toxic analog of Thr. Since HMV is structurally similar to Thr, HNV acts as a feedback inhibitor of AK activity, similar to Thr (28, 29), and becomes incorporated into proteins as a building block, thereby altering the enzyme properties in *E. coli* (30). Most of the biochemical effects of HNV can be recovered by Thr accumulation in the cells. Therefore, yeast mutants that are resistant to HNV are expected to produce large amounts of Thr in the cells, as described previously (10, 27). When the diploid laboratory yeast X2180 (WT) was randomly mutagenized by treatment with ethyl methanesulfonate (EMS), approximately 80 HNV-resistant mutants were obtained. Among these mutants, the strain HNV-5 produced 4.4 times more Thr than the parental strain (16.9 vs 3.8 µmol/g-DCW) when these strains were cultivated in the SD + Am medium (Fig. 2A). In addition, intracellular Met in the strain HNV-5 was increased threefold compared to that of WT, whereas the contents of Asp and Ile in the strain HNV-5 were similar to those of WT (Fig. 2B). Furthermore, although there is no significant difference in Thr content in the culture medium between WT and HNV-5 cells cultivated in a nutrient-rich medium YPD (Fig. S1A), intracellular Thr in the strain HNV-5 was 1.7 times higher than that in the WT strain (Fig. S1B). These results suggest that the strain HNV-5 does not possess mutation(s) for enhancement of Thr uptake and that the increased Thr production is due to mutation(s) of the genes involved in the biosynthesis and degradation of Thr.

### Characterization of mutations in the *HOM3* gene of strain HNV-5

Next, we performed whole-genome sequencing of strain HNV-5 to identify mutation(s) responsible for Thr overproduction. The results showed that this mutant strain had approximately 300 mutations with an amino acid substitution in the genome in comparison to that of the parental X2180 strain. Among the mutated genes, we found a heterozygous mutation, a mixture of guanine and adenine at nucleotide position 1384, in the *HOM3* gene encoding the Ala462Thr variant of Hom3. Ala462 is located in the ACT-2 domain of Hom3, which is responsible for recognizing the inhibitor Thr (Fig. 1B). In addition, the amino acid residue at position 462 is highly conserved as Ala or glycine (Gly) in fungal AKs. This conservation suggests that the amino acid residue corresponding to Ala462 plays a crucial role in allosteric regulation by Thr (Fig. S2). Furthermore, Gly452Asp substitution in the ACT-2 domain decreased sensitivity to Thr feedback inhibition (11, 13). Thus, we hypothesized that the Ala-to-Thr substitution at position 462 also results in release from allosteric regulation by Thr.

### Effect of the Ala462Thr substitution on AK activity and structure of Hom3

**Fig 3:**
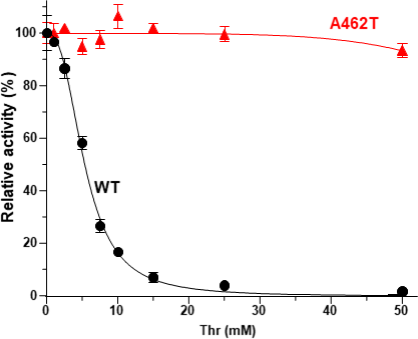
Effect of the Ala462Thr substitution on feedback inhibition of Hom3 by Thr. The relative AK activities of the recombinant WT Hom3 and the Ala462Thr variant (A462T) of Hom3 were measured in the presence of Thr. The enzymatic activities in the absence of Thr are defined as 100% (41.3 U/mg for WT Hom3 and 35.0 U/mg for the A462T variant of Hom3, respectively). Data are presented as means ± standard deviation from three independent experiments.

To evaluate the effect of the Ala462Thr substitution on the AK activity of Hom3, the recombinant Hom3 proteins of WT and the Ala462Thr variant were expressed and purified using *E. coli* cells (Fig. S3). First, the kinetic parameters of the recombinant Hom3 proteins were determined in the absence of Thr. As shown in Table 1, there were no significant differences in kinetic constants (*K*_m_, *k*_cat_, and *k*_cat_/*K*_m_ ratio) between WT Hom3 and the Ala462Thr variant of Hom3, indicating that the Ala462thr substitution had no effect on catalytic ability. Next, we evaluated the sensitivity to Thr feedback inhibition. Thr clearly inhibited the AK activity of WT Hom3 in a dose-dependent manner, and the half-maximal inhibitory concentration (IC_50_) was determined to be 5.4 ± 0.12 mM, in agreement with the previously reported IC_50_ for WT Hom3 (10, 12, 28, 31). Given that the intracellular concentration of Thr in yeast is approximately 4 to 6 mM (32), these data indicate that AK activity of *S. cerevisiae* is subject to feedback inhibition by Thr *in vivo*. In contrast, the Ala462Thr variant was completely insensitive to Thr feedback inhibition even in the presence of 50 mM Thr (Fig. 3).

**TABLE 1:** Kinetic constants of WT and the A462T variant of Hom3^*a*^

Hom3	Aspartate			ATP		
	*K*_m_	*k*_cat_	*k*_cat_/*K*_m_	*K*_m_	*k*_cat_	*k*_cat_/*K*_m_
	(mM)	(s^−1^)	(s^−1^·mM^−1^)	(mM)	(s^−1^)	(s^−1^·mM^−1^)
WT	3.8 ± 0.36	55 ± 1.5	15	0.83 ± 0.044	54 ± 0.78	65
A462T	4.4 ± 0.37	55 ± 1.4	13	1.0 ± 0.071	53 ± 1.1	51

^*a*^ The values are represented as mean ± standard error from three independent experiments.

**Fig 4:**
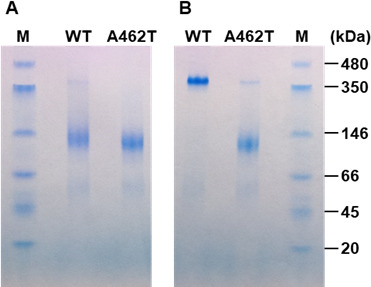
Effect of the Ala462Thr substitution on the conformational change of Hom3 caused by Thr. BN-PAGE of the recombinant Hom3 proteins was carried out in the absence (**A**) and presence (**B**) of Thr. Lanes are as follows: M, molecular mass standards; WT and A462T, and WT Hom3 and the Ala462Thr variant of Hom3, respectively.

**Fig 5:**
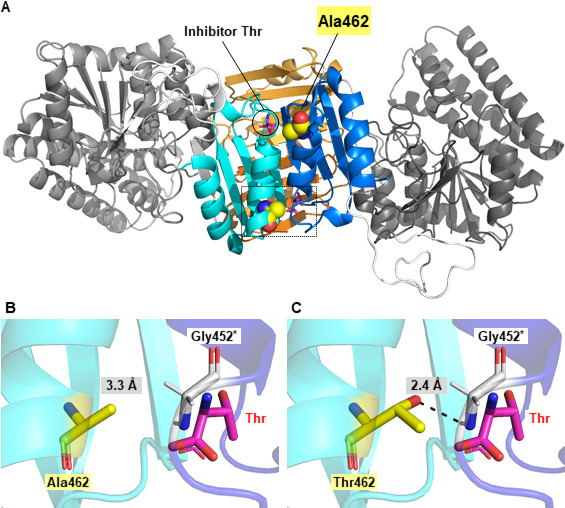
Structural comparison of WT Hom3 with the Ala462Thr variant of Hom3. (**A**) Homodimeric structure of the Hom3 homology model. The contact of two monomers mainly occurred between the ACT domains. The whole protein structure is shown by a cartoon model. The catalytic domain, ACT-1, and ACT-2 in chain A are represented in gray, orange, and cyan, respectively, whereas those in chain B are dark gray, blight orange, and blue. Ala462 is shown as a sphere model in yellow. Two molecules of the inhibitor Thr in *M. jannaschii* AK (MjAK, PDB ID: 3C1N) are superimposed and represented as a stick model in magenta. (**B and C**) Local structure around the residue at position 462 of WT Hom3 (**B**) and the Ala462Thr variant of Hom3 (**C**). The ACT-2 domains of chains A and B are shown in cyan and blue cartoons, respectively. The inhibitor Thr in MjAK is superimposed and represented as a stick model in magenta. The amino acid residues at position 462 [Ala462 and Thr462 in (**B**) and (**C**), respectively] and Gly452 are shown as a stick model in yellow and white, respectively. Values in the gray squares indicate the distance between the side chain of Ala462 or Thr462 and the amino group of the main chain of Gly452. The predicted inter-monomer hydrogen bond between Thr462 and Gly452 is represented as a black dashed line. The asterisk indicates residues from monomer B. Panels (**B**) and (**C**) show a zoomed-in view of the local structure around the residue at position 462 [indicated by the dashed square in (**A**)].

Interestingly, unlike the amino acid substitutions previously reported to reduce sensitivity to Thr feedback inhibition (Table S1) (12, 13), the Ala462Thr substitution did not affect the kinetic constants of Hom3 (Table 1). Structural analyses of AKs from various organisms have shown that binding of the inhibitor alters the conformation of the enzyme, thereby reducing its phosphorylation activity (18–24). Although the crystal structure of the Hom3 protein has not yet been determined, a previous study has indicated that the inhibitor Thr caused an alteration in the apparent molecular mass of the Hom3 homooligomer. Nonetheless, no significant alteration in the apparent mass due to Thr was detected in the feedback inhibition-insensitive Gly452Asp variant (13). This observation implies that the binding of Thr induces a conformational change in the allosteric regulation of Hom3. To evaluate the effect of the Ala462Thr substitution on the conformational alteration by Thr, the oligomeric state of Hom3 proteins in the absence and presence of Thr was investigated by blue-native polyacrylamide gel electrophoresis (BN-PAGE) (33). As shown in Fig. 4A, both WT and the Ala462Thr variant of Hom3 migrated as a band with an apparent molecular mass of approximately 130 kDa in the absence of Thr, suggesting that the Hom3 protein forms a homodimer based on the calculated theoretical molecular mass (60.2 kDa) of a monomer. When 10 mM of Thr was added to both the protein sample and running buffer, the apparent molecular mass of WT Hom3 shifted to approximately 350 kDa, which corresponds to the homohexamer. In contrast, most of the Ala462Thr variant behaved as a homodimer even in the presence of 10 mM Thr (Fig. 4B). These results indicate that the Ala462Thr substitution affects the Thr-mediated conformational changes in allosteric inhibition by Thr. In the homodimeric structure model of Hom3, Ala462 is situated far from the Thr-binding site in the same monomer and establishes no interactions with residues in the Thr-binding site from the neighboring monomer (Fig. 5A and B). Conversely, when Ala462 is replaced with Thr, a hydrogen bond is predicted to form between the hydroxyl group of the side chain of Thr462 and the amino group of the main chain of Gly452 in another monomer (Fig. 5C). One plausible explanation is that this interaction might hinder conformational changes triggered by the inhibitor Thr binding, ultimately resulting in decreased sensitivity to feedback inhibition by Thr. In addition, Gly452 in Hom3 corresponds to a Gly residue that interacts with the inhibitor Thr in the Thr-sensitive AK from *M. jannaschii* bound to Thr (PDB ID: 3C1N) (23). Therefore, it is also plausible that the formation of a putative interaction between Thr462 and Gly452^*^ in the Ala462Thr variant may alter the local conformation of the Thr-binding site, lowering its binding affinity for Thr. The previously reported substitution of Gly452 for Asp also reduced the sensitivity to Thr feedback inhibition and diminished the ability to bind the inhibitor Thr (13). However, the mechanism underlying the decreased sensitivity to feedback inhibition by this amino acid change may be different from that of the Ala462Thr substitution. This is because the Gly-to-Asp substitution at position 452 may form an interaction with Thr456 in the same monomer (Fig. S4). Therefore, further biochemical and structural analyses will be required to elucidate the allosteric regulation mechanism of fungal AKs.

### Effect of Ala462Thr substitution on amino acid productivity

**Fig 6:**
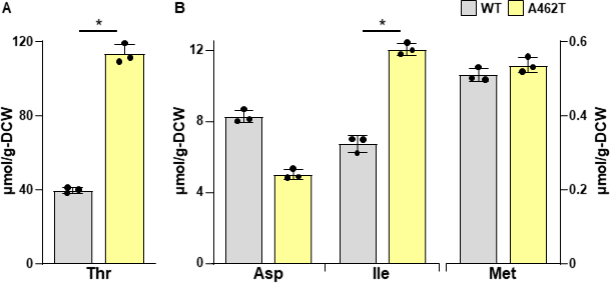
Intracellular amino acid content of yeast cells expressing the *HOM3*^A462T^ gene. Yeast cells were cultured in the SD + Am medium, and the intracellular amino acid content was then measured. Data are presented as means ± standard deviation from three independent experiments. Asterisks indicate statistically significant differences between two strains (Student’s *t* test, *P < 0.05).

The reduced sensitivity of the Ala462Thr variant Hom3 to Thr feedback inhibition suggests that the expression of this variant in yeast increases the intracellular Thr content. We therefore expressed WT and the Ala462Thr variant of Hom3 under the constitutive glyceraldehyde-3-phosphate dehydrogenase (GAPDH) promoter in a *HOM3*-deleted haploid laboratory strain of *S. cerevisiae* (*hom3*Δ cells). When yeast cells were cultivated in minimal SD + Am medium, *hom3*Δ cells expressing the Ala462Thr variant exhibited a 2.8-fold increase in intracellular Thr content compared to WT Hom3 (113 vs 39.9 µmol/g-DCW) (Fig. 6A). Moreover, the expression of the Ala462Thr variant resulted in a 0.6-fold decrease in Asp and a 1.8-fold increase in Ile, relative to cells harboring WT Hom3, while Met content remained virtually unchanged between *hom3*Δ cells expressing WT and the Ala462Thr variant of Hom3 (Fig. 6B). The expression of the *HOM3* genes under the constitutive GAPDH promoter increased the amount of Hom3 protein in *hom3*Δ cells, resulting in higher Thr production (Fig. 6A) than those in X2180-derived strains (Fig. 3A). This is consistent with the findings of a previous study, where overexpression of the *HOM3* gene under an inducible promoter enhanced Thr production (34). Increased Thr productivity probably caused a decrease in Asp content and an increase in Ile content (the former is a precursor of Thr, and the latter is derived from Thr) in *hom3*Δ cells harboring the Ala462Thr variant Hom3 (Fig. 6B). However, this was not observed in strain HNV-5 (Fig. 3B), presumably because the *HOM3* gene was expressed under a native promoter in this strain. On the contrary, strain HNV-5 accumulated more Met than the WT strain (Fig. 2B), but the expression of the Ala462Thr variant Hom3 did not increase the intracellular Met level (Fig. 6B). Given that strain HNV-5 carries approximately 300 mutations, including an amino acid substitution within the Met biosynthetic gene, the phenotypic difference observed between strain HNV-5 and *hom3*Δ cells expressing the Ala462Thr variant Hom3 may be attributable to other gene mutation(s) in strain HNV-5.

### Conclusion

In this study, we isolated the yeast mutant HNV-5, which accumulates Thr in the cells through conventional mutagenesis, and analyzed the mutant *HOM3* gene (*HOM3*^Ala462Thr^) identified in strain HNV-5. The Ala462Thr substitution abolished sensitivity to feedback inhibition by Thr, leading to increased Thr production in yeast cells expressing this Hom3 variant. Moreover, the absence of any impact on the catalytic ability from the Ala462Thr substitution suggests the potential for rational AK engineering to enhance Thr production in yeast even further. These findings will contribute to the development of yeast strains capable of accumulating intracellular Thr. By supplementing EAAs that are deficient in daily diets, this approach holds the potential to extend a healthy lifespan and to improve the quality of life for elderly people.

## MATERIALS AND METHODS

### Strains and culture media

The diploid laboratory yeast strain X2180 (WT, *MAT*a/α) and the haploid laboratory yeast strain BY4741/*hom3*Δ (*hom3*Δ, *MAT*a *his*3Δ1 *leu*2Δ0 *met*15Δ0 *ura*3Δ0 *hom3::KanMX4*) (Horizon Discovery, Cambridge, UK) of *S. cerevisiae* were used as a parent strain for conventional mutagenesis and as a host strain for the expression of the *HOM3* gene, respectively. A nutrient-rich medium YPD (10 g/L yeast extract, 20 g/L peptone, and 20 g/L glucose) and a synthetic dextrose minimal medium SD +Am (1.7 g/L yeast nitrogen base without amino acid and ammonium sulfate (Difco Laboratories, Detroit, MI, USA), 20 g/L glucose, and 5 g/L ammonium sulfate) were used for cultivation of yeast cells. For isolation of HNV-resistant mutants, strain X2180 was grown in the SD +Alt medium (SD medium supplemented with 5 g/L allantoin as a nitrogen source instead of ammonium sulfate).

*E. coli* strains DH5α [F^-^ λ^-^ Φ*80lacZ*Δ*M15* Δ(*lacZYA argF*)*U169 deoR recA1 endA1 hsdR17*(*r_k_*^-^*m_k_*^+^) *supE44 thi-1 gyrA96*] and BL21 (DE3) [F^–^ *ompT hsdS*(r_B_^–^ m_B_^–^) *gal dcm λ*(DE3) (λ(DE3):*lacI*, *lacUV5-T7 gene1 ind1 sam7 nin5*)] were used for construction of the plasmid and for expression of the recombinant Hom3, respectively. *E. coli* strains were cultured in Luria–Bertani (LB) medium (5 g/L yeast extract, 10 g/L tryptone, and 5 g/L NaCl) or TB medium (12 g/L yeast extract, 24 g/L tryptone, 5.02 g/L glycerol, 170 mM KH_2_PO_4_, and 720 mM K_2_HPO_4_) containing appropriate antibiotics. All chemicals were purchased from Wako Pure Chemical (Osaka, Japan), Nacalai Tesque (Kyoto, Japan), and Sigma-Aldrich (St. Louis, MO, USA), unless otherwise stated.

### Construction of expression plasmids for the *HOM3* genes

The DNA fragments of the open reading frame (ORF) encoding WT and the Ala462Thr variant of Hom3 (*HOM3*^WT^ and *HOM3*^A462T^, respectively) were amplified from the genomic DNA of the Thr-accumulating mutant of X2180 (strain HNV-5) by PCR with the primers (5′-GGG GAC AAG TTT GTA CAA AAA AGC AGG CTT AAT GCC AAT GGA TTT CCA ACC-3′ and 5′-A GG GGA CCA CTT TGT ACA AGA AAG CTG GGT GTT AAA TTC CAA GTC TTT TCA-3′). The PCR-amplified DNA fragments were cloned into pDONR221 using BP clonase II, resulting in pDONR_*HOM3*^WT^ and pDONR_*HOM3*^A462T^. To construct the expression plasmids for the recombinant Hom3 proteins, the *HOM3* ORFs were then transferred to the pET53-dest expression vector (Thermo Scientific, Waltham, MA, USA) using LR clonase II (Thermo Scientific), resulting in pET_*HOM3*^WT^ and pET_*HOM3*^A462T^. For construction of the expression plasmids for the *HOM3* genes under the constitutive GAPDH promoter in yeast, the *HOM3* ORFs were transferred into the pAG415_GPD expression vector by the same manner, resulting in pAG415_*HOM3*^WT^ and pAG415_*HOM3*^A462T^. Plasmid pRS416-Cg*HIS3MET15* containing the *URA3*, *HIS3*, and *MET15* genes (35) was used to complement the auxotrophic markers of BY4741/*hom3*Δ cells.

### Isolation of HNV-resistant mutants

Strain X2180 was randomly mutagenized by treatment with 5.5% of EMS in phosphate buffer (pH 7.0) at 30°C for 60 min. EMS-treated cells were washed with 10% (wt/vol) sodium thiosulfate twice and then spread on the SD + Alt medium containing 5 mg/mL HNV. After incubation at 30°C for 2 days, the resulting colonies were collected and tested for amino acid production. The survival rate of yeast cells after EMS treatment was around 40%.

### Quantification of intracellular and extracellular amino acid content

Yeast cells were cultured in the SD + Am medium for 2 days at 30°C and then inoculated into the same medium at an optical density at 600 nm (OD_600_) of 0.1. After cultivation for 48 h at 30°C under shaking, yeast cells were harvested by centrifugation and washed twice. The resulting yeast pellet was resuspended in sterilized water, and the suspension was adjusted to an OD_600_ = 300. Consequently, amino acids in 0.4 mL of the cell suspension were extracted by boiling water at 100°C for 20 min. After centrifugation (5 min at 15,000 × *g*), the amino acid content in each supernatant was subsequently quantified with an amino acid analyzer with ion-exchange chromatography and post-column ninhydrin derivatization (JLC-500/V2, JEOL, Tokyo, Japan). The content of each amino acid was represented as µmol per gram dry cell weight (DCW). In the case of strains WT and HNV-5, we also measured intracellular and extracellular Thr contents of these yeast cells cultivated in the YPD medium as follows. Strains WT and HNV-5 were inoculated into the YPD medium starting from an OD_600_ of 0.1. After incubation for 24 h at 30°C under shaking, yeast cells and the culture medium were separated by centrifugation. Amino acids in the collected cells were extracted using the same method as described above. Thr content in yeast cells and the culture medium was quantified with the amino acid analyzer.

### Whole-genome sequencing

Strains X2180 and HNV-5 were grown in the YPD medium at 30°C for 1 day with shaking. The cells were then harvested, and genomic DNA was extracted by using the Dr. GenTLE (from yeast) High Recovery kit (Takara Bio, Shiga, Japan). Libraries for sequencing analysis were prepared using the NEBNext Ultra DNA Library Prep Kit (New England Biolabs, Ipswich, MA, USA), and paired-end short reads of 150 bp were produced using Illumina NovaSeq 6000 (Illumina, San Diego, CA, USA). The genome sequence of *S. cerevisiae* S288C (https://www.ncbi.nlm.nih.gov/assembly/GCF_000146045.2/) was used as a reference sequence. The sequencing processes were performed via a commercial DNA sequence service (Rhelixa Inc., Tokyo, Japan).

### Expression and purification of the recombinant Hom3 proteins

*E. coli* BL21 (DE3) harboring pET_*HOM3* and pET_*HOM3*^A462T^ were cultivated in TB medium containing ampicillin and grown at 37°C to an OD_600_ of 1.5. The cells were cooled on ice for 5 min, and isopropyl β-d-1-thiogalactopyranoside was added to a final concentration of 0.2 mM. After 20 h of cultivation at 18°C, the cells were harvested by centrifugation and suspended in buffer A [50 mM 4-(2-hydroxyethyl)-1-piperazineethanesulfonic acid (HEPES)-NaOH (pH 7.5), 150 mM NaCl, and 10% (wt/vol) glycerol]. The cell suspension was homogenized under cooling and then centrifuged to remove the insoluble fraction. The supernatant was filtrated by using a 0.45-µm filter and subsequently applied onto a nickel affinity column (Ni Sepharose 6 Fast flow; GE Healthcare, Chicago, IL, USA). After the column was washed with buffer A containing 100 mM imidazole, the recombinant proteins were eluted by buffer A supplemented with 500 mM imidazole.

### Assay of AK activity of the recombinant Hom3 proteins

AK activity was measured by the production of ADP in an enzyme-coupled system with pyruvate kinase (PK) and lactate dehydrogenase (LDH) (12, 13) with modifications. In brief, the reaction mixture (final volume, 1 mL) contained 100 mM HEPES-NaOH (pH 7.5), 40 mM MgSO_4_, 10 mM KCl, 1 mM phosphoenolpyruvate, 0.25 mM NADH, 15 U of PK/LDH (Sigma-Aldrich), 0.5 µg of purified Hom3, and various concentrations of Asp and ATP. The reaction mixture, except for Asp, was pre-equilibrated for 3 min at 30°C, and then the reaction was initiated by the addition of Asp. Hom3-dependent oxidation of NADH was monitored at 340 nm with a DU-800 spectrophotometer (Beckman Coulter, Brea, CA, USA) and maintained at 30°C. For steady-state kinetics, when the concentration of ATP was kept at 5 mM, the concentrations of Asp were varied (1.0–30 mM). With a fixed concentration of 20 mM Asp, the concentration of ATP was 0.25–10 mM. In order to examine the feedback inhibition sensitivity of Hom3, the concentration of Asp and ATP was fixed at 20 and 5 mM, respectively, and Thr was added to the reaction mixture at a concentration of 1.0–30 mM. The reaction rate was calculated with the extinction coefficient of NADH, 6,220 M^−1^‧cm^−1^. One unit of activity was defined as the amount of the enzyme required to produce 1 µmol of ADP per minute. Kinetic parameters of each enzyme were calculated with GraphPad Prism version 9 (GraphPad Software, Boston, MA, USA) using nonlinear regression analysis.

### BN-PAGE analysis

Five micrograms of the purified recombinant Hom3 proteins was loaded on a commercial 5–20% precast poly-acrylamide gel (ATTO, Tokyo, Japan) and electrophoresed at 4°C using native page running buffer (EzRun BlueNative, ATTO) following the manufacturer’s instruction. The native protein marker (EzStandard Native, ATTO) was used for molecular mass estimation. In order to examine the effect of Thr on the oligomeric state of Hom3, the purified proteins were incubated for 5 min at 30°C in the presence of 10 mM Thr and then analyzed with the same method described above, except for the addition of 10 mM Thr to the cathode buffer.

### Structural analysis of the Hom3 protein

The homology model of AK from *S. cerevisiae* [Hom3, (UniProtKB accession no. P10869), homodimer] was downloaded from the SWISS-MODEL repository (36). The template protein used for construction of the model was AK from *M. jannaschii* [32% and 53% sequence identity and similarity to Hom3, respectively (PDB ID no. 3C1N)] (23). The amino acid substitutions of Ala to Thr at position 462 and Gly to Asp at position 452 were introduced using PyMOL software (the PyMOL Molecular Graphics System version 2.5; Schrödinger, LLC). The structure models of WT, Ala462Thr, and Gly453Asp variants of Hom3 were drawn using PyMOL software (http://www.pymol.org).

## Data Availability

The draft genome sequencing data are deposited to the DNA Data Bank of Japan (DDBJ) sequence read archive. The accession numbers of X2180 and HNV-5 are DRR505219 and DRR505220, respectively.

## SUPPLEMENTAL MATERIAL

Table S1, Figures S1 to S4 aem.00155-24-s0001.pdf Supplemental table and figures.

ASM does not own the copyrights to Supplemental Material that may be linked to, or accessed through, an article. The authors have granted ASM a non-exclusive, world-wide license to publish the Supplemental Material files. Please contact the corresponding author directly for reuse.
