# Supplementary material for: Functional analysis of feedback inhibition-insensitive aspartate kinase identified in a threonine-accumulating mutant of Saccharomyces cerevisiae
Source: Appl Environ Microbiol. 2024 Mar 8;90(4):e00155-24. doi: 10.1128/aem.00155-24 (PMC11022571; doi:10.1128/aem.00155-24)
Supplement: Table S1, Figures S1 to S4 — Supplemental table and figures. [file aem.00155-24-s0001.pdf]

Supplemental Material

Functional Analysis of Feedback Inhibition-Insensitive Aspartate Kinase Identified in a Threonine-Accumulating Mutant of *Saccharomyces cerevisiae*

Shota Isogai[1], Akira Nishimura[1], Akiko Inoue[1], Shino Sonohara[2], Takashi Tsugukuni[2], Tomoyuki Okada[2], Hiroshi Takagi[1#]

[1]Institute for Research Initiatives, Nara Institute of Science and Technology, Takayama, Nara, Japan

[2]Plant Bio Business Unit, Musashi Seimitsu Industry Co., Ltd., Toyohashi, Aichi, Japan

Running title: Functional Analysis of Yeast Aspartate Kinase

#Address correspondence to Hiroshi Takagi, hiro@bs.naist.jp

This file includes Table S1 and FIG S1, S2, S3, and S4.

Table S1

Kinetic constants of the previously reported Thr-insensitive variants of Hom3

Hom3	Aspartate			ATP			Reference
	K_m	k_{cat}	k_{cat}/K_m	K_m	k_{cat}	k_{cat}/K_m	
	(mM)	(s^{-1})	($s^{-1}\cdot mM^{-1}$)	(mM)	(s^{-1})	($s^{-1}\cdot mM^{-1}$)	
WT	4.6	47	10	0.74	49	67	12
E279A	2.3	0.98	0.42	1.2	1.1	0.98	
WT	1.2	59 [a]	49[b]	1.2	59 [a]	49[b]	13
G452D	1.1	19 [a]	17[b]	1.2	19 [a]	16[b]	

[a]For comparison to the values in the present study and in reference 12, which are calculated based on the molecular mass of a monomer, the K_{cat} values per monomer are calculated on the basis of those per hexamer in reference 13 (342 for WT Hom3 and 108 for the Gly452Asp variant).

[b]The values are calculated based on the k_{cat} values represented in the table.

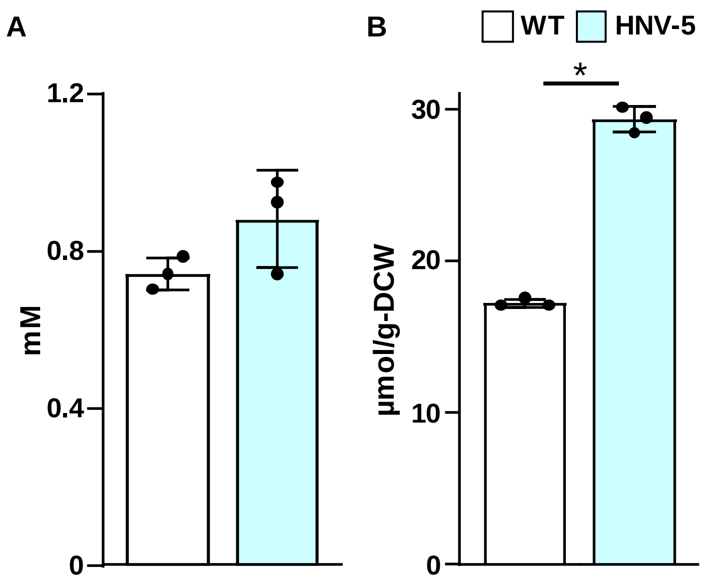

FIG S1 Extracellular (A) and intracellular (B) Thr content of strains WT and HNV-5 cultivated in YPD medium. Yeast cells were cultured in YPD medium, and Thr content in culture medium and yeast cells were then measured. Data are presented as means ± standard deviation from three independent experiments. Asterisks indicate statistically significant differences between two strains (Student's *t*-test, *$p<0.05$).

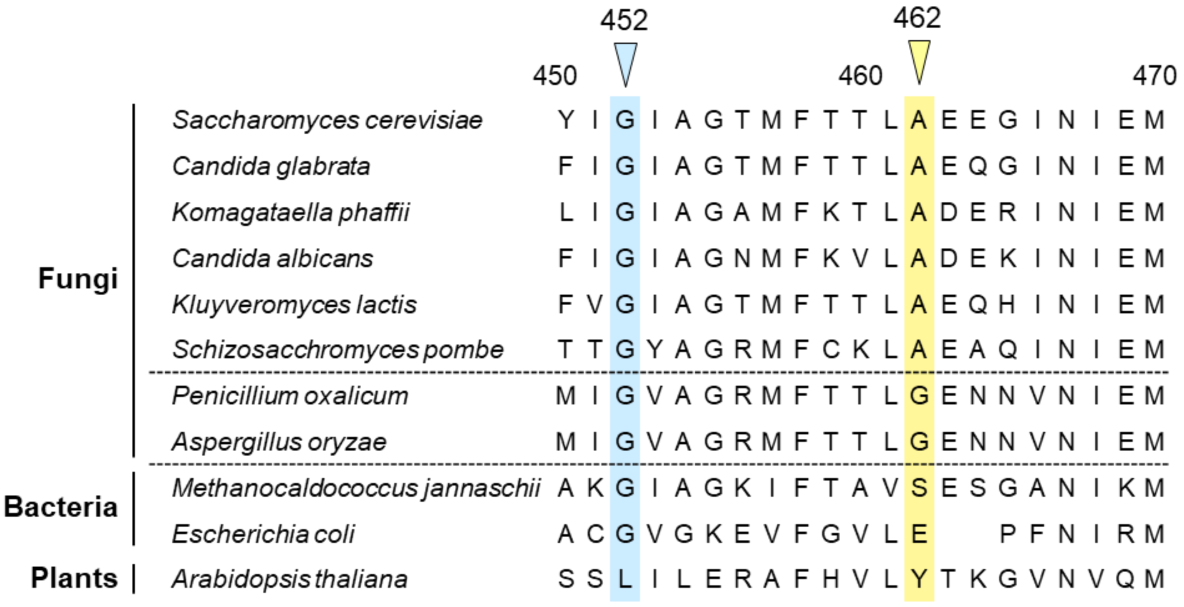

FIG S2 Comparison of amino acid sequences among monofunctional AKs. The amino acid sequence of the ACT-2 domain in the *S. cerevisiae* Hom3 (Uniprot ID: P10869) was compared to those of *Candida glabrata* (Q6FPL5), *Komagataella phaffii* (F2R040), *Candida albicans* (A0A1D8PMB8), *Kluyveromyces lactis* (Q6CID1), *Schizosaccharomyces pombe* (O60163), *Penicillium oxalicum* (S8ANA3), *Aspergillus oryzae* (Q2UTJ9), *Methanocaldococcus jannaschii* (Q58801), *Escherichia coli* (P08660), and *Arabidopsis thaliana* (Q9LYU8) homologues. The *S. cerevisiae* Hom3 and the *M. jannaschii* AK are sensitive to feedback inhibition by Thr, the *E. coli* AK is to that by lysine, and the *A. thaliana* AK is to that by lysine and *S*-adenosyl methionine. Numbering of residues is in Hom3. Ala462 and Gly452 are shown in yellow and light blue, respectively. All sequences are obtained from NCBI and aligned using Crustal omega (https://www.ebi.ac.uk/Tools/msa/clustalo/).

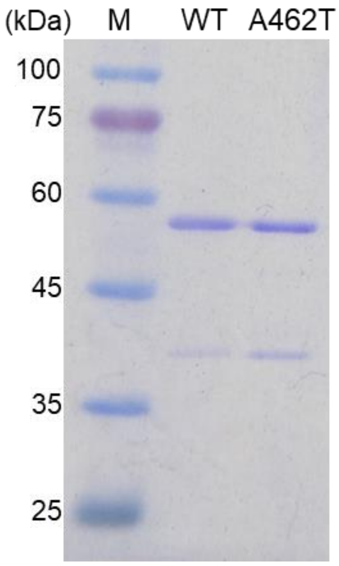

FIG S3 Purification of the recombinant Hom3 proteins. SDS-polyacrylamide gel electrophoresis of the purified recombinant proteins. Lanes are: M, molecular mass standards; WT and A462T, WT Hom3 and the Ala462Thr variant of Hom3, respectively.

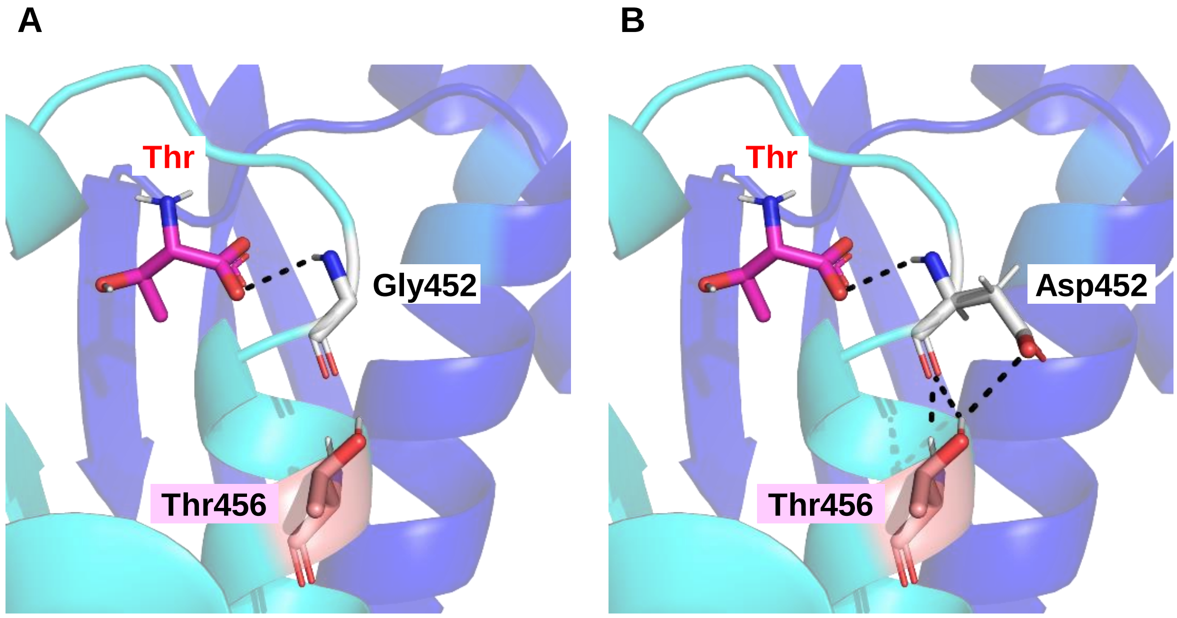

FIG S4 Intra-monomer interactions between Gly452 and Thr456. (A and B) Local structure around the amino acid residues at position 452 of WT Hom3 (A) and the Gly452Asp variant of Hom3 (B) were shown. The ACT-2 domains of chains A and B are shown in cyan and blue carton. The inhibitor Thr is superimposed those in MjAK and represented as a stick model in magenta. The residues at position 452 [Gly452 and Asp452 in (A) and (B), respectively] and Thr456 in chain A are shown as a stick model in white and pink, respectively. The predicted inter-monomer hydrogen bonds between Asp452 and Thr456 are represented black dashed lines.
